# Complete mitochondrial genome of the Small-Branded Swift: *Pelopidas mathias* (Lepidoptera, Hesperiidae)

**DOI:** 10.1080/23802359.2021.1914523

**Published:** 2021-05-04

**Authors:** Xiangyu Hao, Junfeng Zhao, Riwa Hao, Yufei Zhao, Yuan Xiangqun

**Affiliations:** a College of Life Sciences, Northwest A&F University, Yangling, China; b Fujian Key Laboratory of Mineral Resources, Fuzhou University, Fuzhou, China; c Key Laboratory of Plant Protection Resources and Pest Management, Ministry of Education, Entomological Museum, College of Plant Protection, Northwest A&F University, Yangling, China

**Keywords:** *Pelopidas mathias*, mitochondrial genome, phylogeny

## Abstract

We report the complete mitochondrial genome of the Small-Branded Swift: *Pelopidas mathias*, which is an important pest of rice. The total length of the circular double-stranded mitogenome is 15,524 bp, containing 13 protein-coding genes (PCGs), 22 transfer RNAs (tRNAs), 2 ribosomal RNAs (rRNAs) and a non-coding AT-rich region with the nucleotide base composition of 40.07% A, 40.83% T, 11.59% C, and 7.51% G, showing a relatively strong AT bias. The gene order and organization are consistent with typical Lepidoptera species. This work will provide molecular data support for the study of the phylogeny and evolution in the family Hesperiidae.

The skipper *Pelopidas mathias* Fabricius 1798, commonly known as the Small-Branded Swift, is a representative species of in the family Hesperiidae. *P. mathias* and its sister species *P. agna* Moore, live in a mixture in some places (Lee 1966), and are often considered as the major pests of the cereal crops (*Oryza sativa*, *Zea mays*, *Sorghum vulgare*, etc) in China. The larvae of this species feed on rice and cause great harm to the crops production (Yuan et al. 2014; Yuan et al. 2015).

In this study, we sequenced, assembled and annotated the complete mitochondrial genome of *Pelopidas mathias* (GenBank accession number MW264491), and compared it with other mitogenomes of hesperids available (Li et al. 2019), aiming to further clarify its phylogenetic relationship with other Hesperiidae species. The specimen was collected at Lushan Mountain (Jiujiang, Jiangxi Province, China) (Geodetic Coordinate: g115.994489, 29.555792) in August 2016. The sample (NWAFU-PPC-YuanLab20160827) was stored in the Entomological Museum of Northwest A&F University (URL: https://ppc.nwafu.edu.cn/english/aboutus/index.htm; Contact person: Xiangqun Yuan, yuanxq@nwsuaf.edu.cn). Genomic DNA was extracted using Genomic DNA Kit (TransGen Biotech, Beijing) and sequenced on an Illumina HiSeq 2000 platform (Biomarker Technologies, Beijing). Each Illumina HiSeq read was 150 bp and the 1.2 Gb raw data was trimmed with default parameters, then clean reads were preliminarily assembled using *de novo* assembly in the CLC Genomics Workbench v10.0.1 (CLC Bio, Aarhus, Denmark). The various genomic features were annotated using Geneious 8.1.3 referenced to the complete mitogenome sequence of *Polytremis nascens* (Jiang et al. 2016) and *Parnara guttatus* (Shao et al. 2015) available from GenBank.

The genome is 15,524 bp in size and contains 13 protein-coding genes (PCGs) (*nad1*, *nad2*, *cox1*, *cox2*, *apt8*, *atp6*, *cox3*, *nad3*, *nad4L*, *nad4*, *nad5*, *nad6*, and *cytb*), 2 rRNA genes (*rrnS* and *rrnL*), 22 tRNA genes (*trnM, trnI, trnQ, trnW, trnC, trnY, trnL2, trnK, trnD, trnG, trnA, trnR, trnN, trnS1, trnE, trnF, trnH, trnT, trnP, trnS2, trnL1, trnV*), and a non-coding AT-rich region (D-loop region), with its gene order and organization similar to those of most other butterflies (Park et al. 2016). Like the common features of insects (Boore 1999), the genome shows a relatively strong AT bias with a base composition of 40.07% A, 40.83% T, 11.59% C, and 7.51% G. The 13 PCGs are totally 11,175 bp in size, encoding 3,725 amino acids. The *nad5*, *nad4*, *nad4L* and *nad1* genes are encoded in L-strand and the other PCGs are encoded in H-strand. All PCGs terminate with the stop codon TAA expect for *cox1*, *cox2*, *nad5* and *nad4* (using TNN).

The 22 tRNA genes together comprise 1,460 bp in size, and all of them can be folded into a cloverleaf secondary structure, with the exception of *trnS1* lacking the ‘DHU’ arm. The *rrnS* and *rrnL* are 791 bp and 1,379 bp long, respectively. The AT-rich region located between *rrnS* and *trnM* is the longest non-coding region in the entire mitochondrial genome sequence, with a high A + T content of 93.98%.

The phylogenetic relationships of 37 species (including 33 Hesperiidae species and 4 Papilionidae outgroups) were reconstructed with Bayesian inference (BI) (Ronquist et al. 2012) methods based on the PRT datasets (concatenating 13 PCGs, 22 tRNAs and 2 rRNAs). All nodes have a high posterior probability (Figure 1). The result showed that *P. mathias* is sister group to *Parnara guttatus*.

**Figure 1.**
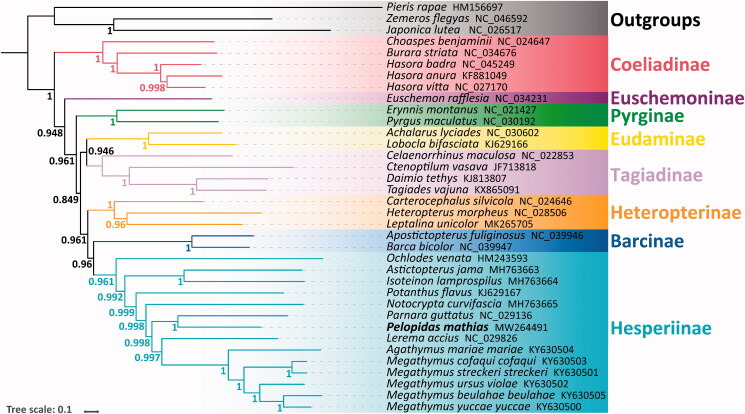
The phylogenetic relationships of *Pelopidas mathias* inferred by BI method based on PRT dataset.

## Data Availability

The following information was supplied regarding the availability of DNA sequences: The complete mitogenome of *Pelopidas mathias* is deposited in GenBank of NCBI at https://www.ncbi.nlm.nih.gov/nuccore/1967016211, accession number MW264491. The associated BioProject, SRA, and Bio-Sample numbers are PRJNA707666, SRR13906282, and SAMN18219118 respectively.
